# Variations in Tobacco Retailer Type Across Community Characteristics: Place Matters

**DOI:** 10.5888/pcd19.210454

**Published:** 2022-08-11

**Authors:** Claire Jenkins, Elli Schwartz, Nathaniel Onnen, Peter F. Craigmile, Megan E. Roberts

**Affiliations:** 1 College of Public Health, The Ohio State University, Columbus, Ohio; 2 Department of Statistics, The Ohio State University, Columbus, Ohio

## Abstract

**Introduction:**

The density of tobacco retailers varies by community characteristics such as poverty levels or racial and ethnic composition. However, few studies have investigated how specific types of tobacco retailers vary by community characteristics. Our objective was to assess how the types of tobacco retailers in Ohio varied by the characteristics of the communities in which they were located.

**Methods**

All census tracts in Ohio were coded for 3 characteristics: poverty (high or low), prevalence of racial and ethnic minorities (high or low), and urban, suburban, or rural status. We then coded all 11,392 tobacco retailers in the state for its retailer type category. Finally, we tested whether the prevalence of particular types of tobacco retailers varied by particular types of census tracts.

**Results:**

For all US Census tracts, convenience stores were the most common type of retailer selling tobacco. Yet, the prevalence of convenience stores was higher in high-poverty urban tracts than in low-poverty urban tracts. Discount stores were the second-most common type of tobacco retailer and were most prevalent in rural tracts and high-racial and ethnic minority urban tracts. Grocery stores, pharmacies, and vape or hookah shops typically had the highest prevalence in more advantaged tracts.

**Conclusion:**

Our findings demonstrate that the distribution of specific retailer types varies by community characteristics. The distribution of these retailer types has implications for product availability and price, which may subsequently affect tobacco use and cessation. To create equitable outcomes, policies should focus on retailers such as convenience and discount stores, which are heavily located in communities experiencing tobacco-related health disparities.

**Summary**

**What is already known on this topic?**

A strong body of research indicates that the density of tobacco retailers varies by community characteristics.

**What is added by this report?**

In Ohio, the distribution of tobacco retailer types also varied by community characteristics. Convenience stores and discount stores selling tobacco were more common (whereas grocery stores and pharmacies were less common) in historically disadvantaged US Census tracts, based on poverty, racial and ethnic composition, or urban and rural status.

**What are the implications for public health practice?**

Policies to reduce tobacco retailer density should focus on retailers such as convenience and discount stores, which are heavily located in communities experiencing tobacco-related health disparities.

## Introduction

Numerous studies show that a higher density of tobacco retailers (ie, any type of store that sells tobacco, including convenience stores, pharmacies, and tobacco shops) is associated with a higher likelihood of tobacco use among both youth and adults (1–6) and a lower likelihood of cessation success (7). Of added concern is the finding that retailer density varies greatly by community characteristics. Specifically, neighborhoods with higher proportions of racial and ethnic minorities and people of lower socioeconomic status (SES) tend to have higher densities of tobacco retailers, compared with more advantaged neighborhoods (8–10). Findings regarding urban versus rural disparities are mixed, with some studies reporting higher per capita retailer density for urban areas (8) and others for rural areas (11).

Although these general disparities in tobacco retailer density are well established in public health literature, few US studies have investigated how particular types of tobacco retailers vary by community characteristics. In other fields outside of tobacco research, grocery stores have been found most available in suburban areas and least available in rural areas, whereas convenience stores are most prevalent in urban, low-income areas (12). Other studies have separately found differences in the distribution of liquor stores and bars (13), discount and dollar stores (14,15), vape shops (16,17), pharmacies (18), and tobacco shops (18). Although these studies are a start, we need a comprehensive understanding of how various types of tobacco retailers are distributed. Understanding these patterns is critically important, as the distribution of specific types of retailers (eg, discount stores, convenience stores, vape shops) could contribute to tobacco-related disparities by affecting factors such as tobacco price, product availability, and retailer licensing. Therefore, our objective was to assess how tobacco retailer type varied by community characteristics.

## Methods

We assessed how tobacco retailer type in Ohio varied by community characteristics — poverty level, prevalence of racial and ethnic minorities, and urban, suburban, and rural status — in 2017. With a population of over 11.6 million residents, Ohio is an ideal setting for this research because of its varied sociodemographic profile containing wide ranges in income level, diverse urban centers (eg, Columbus, Cleveland, Cincinnati), and highly rural areas (including 32 Appalachian counties). At the time of data collection, Ohio also lacked any licensing policies that could influence market-driven distributions in retailer type (19).

### Tobacco retailer data

Ohio law requires retailers to obtain a license to sell cigarettes, although a license is not required for selling other tobacco products, such as e-cigarettes. Names and addresses of all retailers with active cigarette licenses (eg, convenience stores, tobacco shops) are publicly available in Ohio and were obtained from each of Ohio’s 88 county auditor offices in the fall of 2017. For other types of tobacco retailers that do not require a cigarette license (ie, hookah cafés and vape shops), we used established research methods for searching internet directories (20) (eg, Yelp.com, Yellowpages.com). Our final list consisted of 11,392 tobacco retailers (11,065 cigarette retailers and 327 vape and hookah shops). These retailers were then geocoded based on their street address. Details are provided elsewhere (11) on our process for obtaining retailer data, cleaning and duplicate removal, telephone-based validation of a random sample, and geocoding with the R statistical package (R Foundation).

### Coding for retailer type

With the names and addresses of all 11,392 tobacco retailers in Ohio, we next coded each for retailer type. We assigned all retailers to 1 of 9 retailer categories: convenience store, discount store, grocery store or mass merchandiser, pharmacy, bar or restaurant, tobacco shop, alcohol store, vape or hookah shop, or other (Table 1). These 9 categories were drawn from STARS (21), a validated retailer surveillance tool, which we modified for our purposes; in particular, categories were split to examine retailers of interest, such as discount stores. Vape and hookah retailers (a combined category) were already identified at the time of data collection, as previously described. For pharmacies, we obtained a list of all licensed pharmacies with brick-and-mortar locations in fall 2017 from the State of Ohio Board of Pharmacy. We then matched this list with our list of tobacco retailers, because not all pharmacies sell tobacco. In instances where a pharmacy was located within a larger business site, the retailer was coded based on the larger business site (eg, a grocery store that contained a pharmacy was coded as a grocery store, not a pharmacy). For the remaining retailers, keyword searches in the retailer’s name were used to classify retailer types, similar to methods used by others (22,23). Keywords included names of large chains (eg, Walmart, Speedway, Dollar General) and other indicators of retailer type (eg, grill, liquor, delicatessen). Retailers for which type could not be definitively identified through keywords were researched individually, based on their names and addresses.

**Table 1:** Number and Prevalence of Retailer Types Among All Tobacco Retailers in Ohio (N = 11,392), 2017

Retailer Type	Number	Prevalence, %	Definition	Sample retailer names
Convenience stores	6,438	56.5	Gas stations, corner stores, drive-through stores, and any stand-alone store stocking a limited supply of foods or household goods	Speedway, BP, Stop & Go
Discount stores	1,229	10.8	Stores selling goods at a lower cost than the typical market value	Dollar General, Family Dollar
Grocery stores and mass merchandisers	1,005	8.8	Stores stocking a large supply of fresh produce, meat, and dairy products or a wide variety of household goods across multiple product categories	Kroger, Giant Eagle, Walmart, Sam’s Club
Pharmacies	610	5.4	Stores registered with a pharmacy license	Rite Aid, Walgreens, Discount Drug Mart
Bars and restaurants^a^	642	5.6	Establishments serving food and drinks or selling prepared meats	Westlake Tavern, Laila’s Pizza, Columbus Beverage & Deli
Tobacco shops	361	3.2	Stores that primarily sell tobacco in the form of cigars or cigarettes	Just Smokes, Cheap Tobacco
Alcohol stores^a^	348	3.1	Stores that primarily sell alcohol including state liquor stores and wine cellars	Discount Liquors, Cleveland Wine and Beverage
Vape and hookah shops	327	2.9	Tobacco retailers that primarily sell hookah and e-cigarettes	Kings of Vapor, Vapor Station, Waterbeds ‘n’ Stuff
Other	432	3.8	Retailers that did not match any of the defined categories	Pilot Travel Center, Scioto Downs Casino, Valley Golf Course

a Most retailers in this category were independent stores. The names of sample stores have been altered to prevent the identification of specific retailer locations.

### Community characteristics

We classified communities at the census tract–level. For all Ohio census tracts, we obtained information about poverty, race, and ethnicity from the 2016 American Community Survey 5-Year Estimates (2,951 census tracts, after the removal of 1 tract that was missing poverty information). Given the skewed distributions of these variables, we chose to dichotomize them into low and high to simplify the interpretation of our findings. Census tracts were coded as high prevalence of poverty if more than 15.4% of the population was below the poverty level (15.4% was the state average for Ohio); all other tracts were coded as low prevalence of poverty. If 15% or more of the population was Non-Hispanic Black (Black) or Hispanic, tracts were coded as high prevalence Black or Hispanic residents. Other tracts were coded as low prevalence of Black or Hispanic residents. Other racial and ethnic groups were not included because of their low prevalence in Ohio (eg, only 1.3% of tracts had a high prevalence of Asian residents) (11). All cut-offs distinguishing high and low groups were selected a priori and are consistent with our previous work (11,24).

To determine whether a tract was urban, suburban, or rural, we used the county-level classifications applied by the Ohio Family Health Survey, now the Ohio Medicaid Assessment Survey (25). This system classifies all Ohio counties as either metropolitan (urban), suburban, rural non-Appalachian, or rural Appalachian. For analyses, we combined the 2 rural designations.

### Data analysis

Analyses began with descriptive statistics to determine the prevalence of the various tobacco retailer types in Ohio overall. A series of 2 × 2 χ^2^ analyses were then conducted to determine whether the prevalence of a particular tobacco retailer type (eg, prevalence of retailers that were convenience stores versus prevalence of retailers that were not convenience stores) varied between 2 census tract types. These χ^2^ analyses were conducted for all retailer types and pairs of census tract types with sufficient data. To increase cell sizes, the variables for prevalence of Black and Hispanic populations were combined to create an overall racial and ethnic minority prevalence variable (where high minority indicated high prevalence of Black or Hispanic populations, and low minority indicated low prevalence of Black and low prevalence Hispanic populations). Yet, some census-tract types were still very rare in the state and, therefore, had a very low prevalence of retailers. For this reason, high racial and ethnic minority census tracts in the suburban and rural parts of Ohio did not have sufficient data (<1% of all retailers) and were excluded from analyses. Because of its varied composition, the retailer category “other” was also excluded from analyses. Ultimately, we compared 8 retailer types and 8 census tract types. Because of the large number of statistical tests performed, we adjusted for the overall false discovery rate by using the Benjamini-Hochberg procedure (26).

## Results

The overall prevalence of tobacco retailer types in Ohio are presented (Table 1). The prevalence of tobacco retailer type within each tract type are shown in (Table 2) and (Figure). For all census tracts, convenience stores were the most common type of retailer selling tobacco. For the state overall, 56.5% of tobacco retailers were convenience stores. However, the prevalence of convenience stores varied by community type: they were more prevalent in high-poverty, urban, racial and ethnic minority census tracts, as well as in high-poverty, urban, nonminority census tracts. No differences were observed in convenience store prevalence by poverty in suburban or rural census tracts (Table 2) (Figure).

**Table 2:** Prevalence of Tobacco Retailer Types Within Each Ohio Census Tract Type, 2017

Retailer type	Census tract type							
	High minority, high poverty	High minority, low poverty	Low minority, high poverty	Low minority, low poverty	Low minority, high poverty	Low minority, low poverty	Low minority, high poverty	Low minority, low poverty
	Urban (%)				Suburban (%)		Rural (%)	
Convenience stores	62.6^a^	52.6^b^^,^ c	59.1^a^^,^ b	54.9^c^	58.5^a^^,^ b ^,^ c ^,^ d	59.9^a^^,^ d	59.1^b^^,^ d	60.1^a^^,^ d
Discount stores	11.8^a^^,^ b	12.0^a^^,^ b ^,^ c ^,^ d	8.8^c^	8.6^c^	12.7^a^^,^ b ^,^ d	9.4 a ^,^ c	14.5^d^	13.3^b^^,^ d
Grocery stores and mass merchandisers	6.9^a^	10.9^b^^,^ c	8.1^a^^,^ b ^,^ d	10.4^c^	7.8^a^^,^ b ^,^ c	10.8^c^	8.9^c^^,^ d	10.2^b^^,^ c ^,^ d
Pharmacies	4.3^a^^,^ b	8.0^c^^,^ d	5.0^a^^,^ b ^,^ c	8.4^d^	5.2^a^^,^ b ^,^ c	5.0^a^^,^ b ^,^ c	5.1^a^^,^ b ^,^ c	3.6^b^
Bars and restaurants	7.4^a^	6.9^a^	7.5^a^	5.9^a^	2.4^b^	4.0^b^	4.0^b^	6.7^a^
Tobacco shops	1.9^a^	3.3^a^^,^ b	4.5^b^	3.7^b^^,^ c	5.2^b^	2.7 a ^,^ c	4.0^b^^,^ c	2.2^a^
Alcohol stores	3.5^a^	4.0^a^^,^ b	3.4^a^	3.8^a^	4.2^a^	4.4^a^	2.2^b^^,^ c	1.8^c^
Vape and hookah shops	1.6^a^	2.2^a^^,^ b	3.7^b^	4.2^b^	4.0^b^	3.9^b^	2.2^a^	2.1^a^

a Results based on χ^2^ tests. Superscripts are by row and indicate whether prevalence of retailer type differs by census tract type. Within a row, values that do not share a superscript are significantly different from one another. Conversely, within a row, values that share a superscript do not significantly differ.

b Indicates all values in the row do not significantly differ.

c Indicates all values in the row do not significantly differ.

d Indicates all values in the row do not significantly differ.

**Figure:**
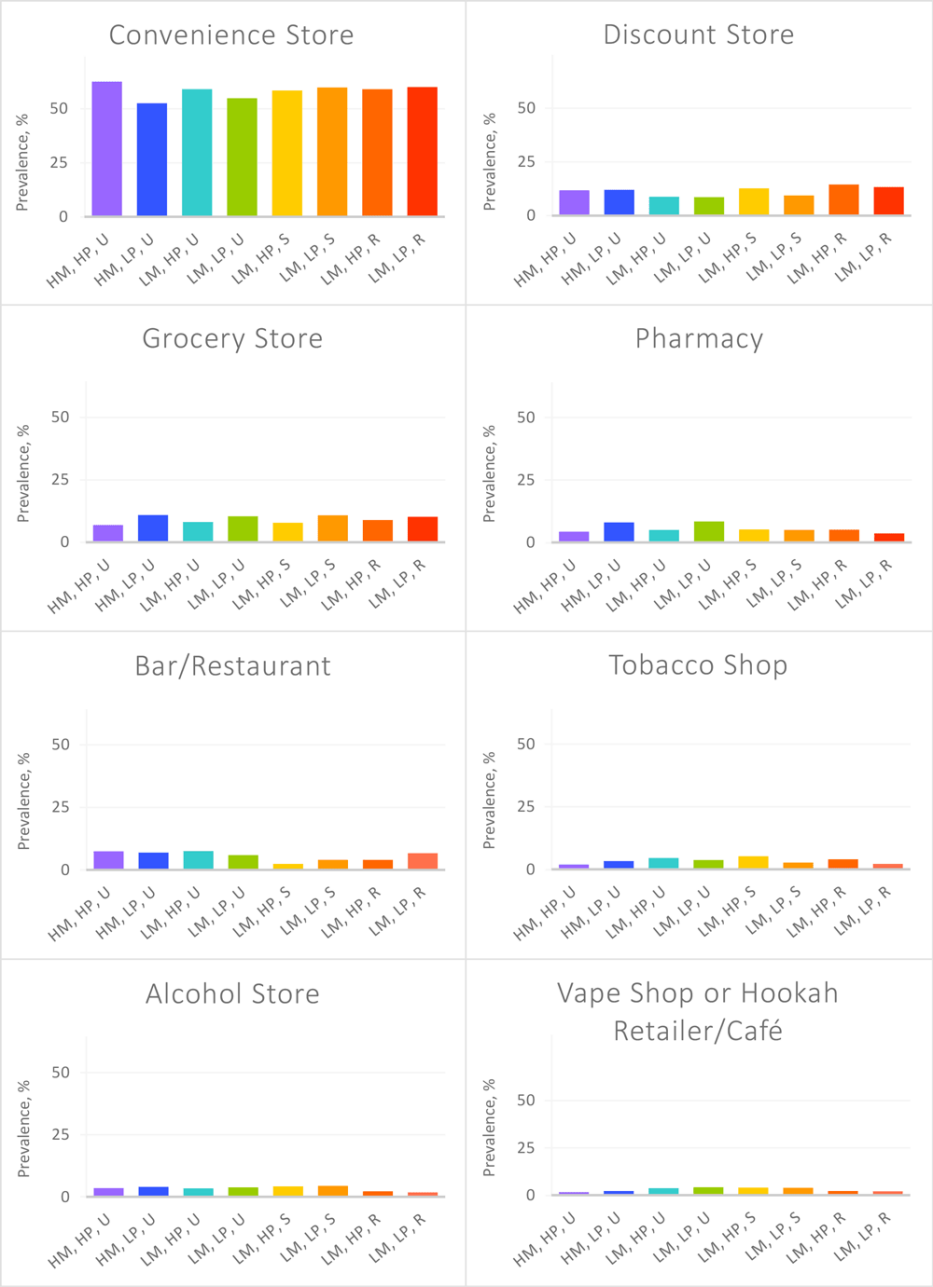
Prevalence of tobacco retailer type within each type of census tract. Abbreviations: HM, high minority; HP, high poverty; LM, low minority; LP, low poverty, R, rural; S, suburban; U, Urban.

**Table:** 

Retailer type	High minority, high poverty, urban	High minority, low poverty, urban	Low minority, high poverty, urban	Low minority, low poverty, urban	Low minority, high poverty, suburban	Low minority, low poverty, suburban	Low minority, high poverty, rural	Low minority, high poverty, rural
Convenience	62.6	52.6	59.1	54.9	58.5	59.9	59.1	60.1
Discount	11.8	12	8.8	8.6	12.7	9.4	14.5	13.3
Grocery	6.9	10.9	8.1	10.4	7.8	10.8	8.9	10.2
Pharmacy	4.3	8	5	8.4	5.2	5	5.1	3.6
Bar, restaurant	7.4	6.9	7.5	5.9	2.4	4	4	6.7
Tobacco shop	1.9	3.3	4.5	3.7	5.2	2.7	4	2.2
Alcohol store	3.5	4	3.4	3.8	4.2	4.4	2.2	1.8
Vape shop or hookah retailer/cafe	1.6	2.2	3.7	4.2	4	3.9	2.2	2.1

As the second-most common type of tobacco retailer overall, discount stores also showed disparities in their distribution. Discount stores were significantly more common in high-racial and ethnic minority/high-poverty urban census tracts, high-poverty suburbs, and both low- and high-poverty rural census tracts than in low-racial and ethnic minority urban areas, which had the lowest prevalence.

Grocery stores demonstrated the opposite poverty-based pattern as convenience stores. They were less prevalent in high-poverty (vs low-poverty) urban, racial and ethnic minority census tracts, as well as for high-poverty (vs low-poverty) low racial and ethnic minority urban census tracts. Although there was a trend in the same direction for suburban and rural census tracts having lower prevalence in high-poverty census tracts, the differences were not significant. Similar patterns were found for pharmacies. The prevalence of pharmacies was nearly half the rate for high-poverty (vs low-poverty) urban census tracts with high prevalence of racial and ethnic minorities. No differences were observed in pharmacy prevalence by poverty within suburban or rural census tracts.

Bars and restaurants selling tobacco products were more prevalent in urban and low-poverty census tracts than in suburban and high-poverty rural census tracts. Tobacco shops were more common in low-racial and ethnic minority, high-poverty urban and suburban census tracts than high-racial and ethnic minority, high-poverty urban census tracts or low-racial and ethnic minority, low-poverty suburban and rural census tracts. Alcohol store prevalence was lower in rural census tracts than in nearly all other census tract types. Finally, vape and hookah shops had the lowest prevalence in high-racial and ethnic minority, high-poverty urban census tracts and rural census tracts.

## Discussion

Our study examined disparities in the distribution of tobacco retailer types by census tract poverty level, racial and ethnic composition, and urban, suburban, and rural status. One of the clearest patterns that emerged was the poverty-based disparities in urban areas. For urban centers in Ohio in areas of both high- and low- racial and ethnic minority areas, the prevalence of convenience stores was higher in high-poverty census tracts than in low-poverty tracts. Grocery stores and pharmacies demonstrated the opposite pattern as convenience stores, whereby, for urban centers and areas of both high- and low- racial and ethnic minority areas, the prevalence of grocery stores and pharmacies was lower in high-poverty census tracts than in low-poverty tracts. These findings align with previous studies (12).

Disparities in the distribution of discount stores also appeared to be driven by racial and ethnic composition. Low-minority urban tracts had the lowest prevalence of discount stores, and high-minority urban tracts had among the highest prevalence of discount stores. This finding aligns with arguments by others that the growth of discount stores in urban neighborhoods of color relates to a long history of racial discrimination and economic exclusion (15). A high prevalence of discount stores was also found in high-poverty suburbs, and consistent with previous work, in both low-poverty and high-poverty rural census tracts (14). Tobacco shops showed a somewhat different pattern, such that low racial and ethnic minority urban census tracts had some of the highest prevalence of tobacco shops. A high prevalence of tobacco shops was also found in high-poverty suburban and rural census tracts.

Finally, some disparities in prevalence of various types of tobacco retailers were driven by differences in urban, suburban, or rural status. Both vape shops, hookah shops, and alcohol stores had the lowest prevalence in rural census tracts, consistent with a previous study on vape shops (16). That same study however, found vape shops to be more concentrated in Hispanic and Asian areas, whereas our data showed vape and hookah shops as low-prevalence in high-minority urban tracts. This discrepancy could be a result of different classifications of racial and ethnic minorities in the studies. Another study, which did not test for urban and rural differences, found lower vape shop density in New Jersey census tracts with a higher proportion of Hispanic and Black residents (27). We also found bars and restaurants that sold tobacco were least prevalent in suburban and rural census tracts than in urban tracts, with the exception of low-poverty rural tracts.

Our study builds on previous research on tobacco retailer disparities (8,11) by showing disparities in the distribution of specific retailer types. Our findings are meaningful because of their implications for product price and availability. For example, because discount stores are heavily concentrated in minority, rural, and high-poverty census tracts, people living in these communities have access to tobacco at cheaper prices. A recent study found nearly a one-dollar difference in the price of cigarette packs at discount stores than at grocery stores (28). The distribution of discount stores, therefore, likely contributes to sustaining the high prevalence of tobacco use among racial and ethnic minority, rural, and high-poverty populations. This prospect is further concerning given the evidence that, among all tobacco retailers in the US, the number of discount stores is disproportionately increasing (29).

Another finding was that vape and hookah shops have the lowest prevalence in high-minority, high-poverty urban tracts and rural tracts. Consequently, these communities may have less access to e-cigarettes and hookah tobacco. The public health effect of this finding is unclear. Complex debates continue regarding e-cigarettes, including their role as potential harm reduction products for adult cigarette smokers looking to quit and as potential gateway products for youth who might have not otherwise initiated tobacco use (30–32). The consequences of vape shop access for health disparities will need to be examined in future research.

Our findings also have implications for a community’s selection of licensing-law strategies. Licensing laws are policies that require retailers to obtain a license to sell tobacco; licensing-law strategies can set additional stipulations aimed at reducing or restricting the number or density of retailers in an area (33). For example, several localities have used licensing-law strategies that prohibit the sale of tobacco in pharmacies. Our data, however, show pharmacies are most prevalent in low-poverty urban census tracts, contributing to what some have called pharmacy deserts (34,35). This type of distribution suggests that policies restricting pharmacy tobacco sales could have an inequitable effect by eliminating more retailers in the most affluent areas. This equity concern has been raised by other researchers (36). Conversely, as our data show both convenience stores and discount stores are more prevalent in communities experiencing tobacco-related health disparities, these retailer types should be the target of future policies to equitably reduce tobacco retailer density. For instance, an age-restricted in-person location policy could limit the sale of tobacco products to adult-only locations, limiting tobacco retailers to tobacco, vape, and hookah shops, or state-run outlets. Age-restricted in-person location policies mirror how cannabis and liquor sales are restricted in some states. Licensing-law strategies can also equitably reduce retailer density, when thoughtfully selected on the basis of community characteristics (24).

Our study had limitations. At the time of data collection, Ohio only required a license for selling cigarettes, and no licensing-law strategy existed in the state. Although that licensing system provided a seemingly comprehensive list of all cigarette retailers, it is possible that some cigarette retailers were unlicensed. It is also possible that our search methods might not have detected all hookah and e-cigarette retailers. Another limitation is that Black and Hispanic census tracts were combined, limiting our understanding of more granular community-level differences in the distribution of retailer types. As analyses for this study were limited to Ohio, future research should replicate our approach in other areas of the US that have different distributions of community characteristics. Future research could also analyze additional retailer categorizations (eg, chain vs nonchain retailers).

Our findings demonstrate that the distribution of specific retailer types vary by community characteristics. We found that grocery store and pharmacy distributions varied by community poverty, discount stores and tobacco shops varied by poverty and racial and ethnic composition, and vape, hookah, and bars and restaurants varied by urban, suburban, or rural status. The distribution of these retailer types has implications for product availability and price, which may subsequently influence tobacco use and cessation. To create equitable outcomes, policies should focus on retailers, such as convenience and discount stores, which are heavily located in communities experiencing tobacco-related health disparities.
